# Black Soldier Fly Larvae’s Optimal Feed Intake and Rearing Density: A Welfare Perspective (Part II)

**DOI:** 10.3390/insects16010005

**Published:** 2024-12-26

**Authors:** Arianna Cattaneo, Simona Belperio, Luca Sardi, Giovanna Martelli, Eleonora Nannoni, Sihem Dabbou, Marco Meneguz

**Affiliations:** 1 Center Agriculture Food Environment (C3A), University of Trento, 38098 San Michele All’Adige, Italy; arianna.cattaneo@unitn.it; 2 Department of Veterinary Sciences (DIMEVET), University of Bologna, 40064 Ozzano dell’Emilia, Italy; simona.belperio2@unibo.it (S.B.); luca.sardi@unibo.it (L.S.); giovanna.martelli@unibo.it (G.M.); eleonora.nannoni2@unibo.it (E.N.); 3 BEF Biosystems s.r.l., 10156 Torino, Italy; marco.meneguz@bef.bio

**Keywords:** black soldier fly, insect rearing, feeding rate, rearing density, welfare

## Abstract

The large-scale insect rearing sector is expected to grow significantly in the next few years, with *Hermetia illucens* L. (black soldier fly, BSF) playing a pivotal role. As with traditional livestock, it is essential to improve and ensure BSF welfare. A starting point can be an adaptation of the Five Freedoms framework. Feed availability must be optimized to meet larvae nutritional needs (freedom from hunger) while maximizing substrate conversion efficiency. Similarly, rearing density needs to be optimized to ensure well-being, particularly in large-scale operations. In this study, Control (commercial laying hen feed) and Omnivorous substrates (vegetable and meat) were used as dietary regimes. In the first trial, three feeding rates were tested: 50, 100, and 200 mg feed/larva/day; in the second trial, three rearing densities were evaluated: 5, 10, and 15 larvae/cm^2^. Performance parameters, including final larval weight, final frass biomass, growth rate, substrate reduction, feed conversion ratio, larval length, survival rate, larvae chemical composition, and process optimization, were studied. Our results show that a feeding rate of approximately 90 mg feed/larva/day in the Omnivorous diet and 175 mg feed/larva/day in the Control diet, along with a rearing density of 5 and 7.57 larvae/cm^2^, respectively, in the Omnivorous and Control diets, produced optimal growth performances ensuring larval well-being. This outcome offers valuable insights for implementing good welfare practices in the insect farming sector and optimizing rearing management and efficiency.

## Simple Summary

The lack of welfare guidelines in the insect rearing sector is an aspect of growing concern. This study investigates the larval stage of the black soldier fly (L.) (Diptera: Stratiomyidae), examining how different feeding rates and densities during the rearing period impact their welfare, applying dietary regimes already validated in previous research. Feeding rates of 50, 100, and 200 mg feed/larva/day and densities of 5, 10, or 15 larvae/cm^2^ were studied. By using performance parameters as indirect indicators of welfare, guided by the Five Freedoms framework and applying performance optimization models, the results showed that in the Omnivorous diet, a medium feeding rate (90 mg/larva/day) and density of 5 larvae/cm^2^ led to optimal performances without providing excessive feed or causing overcrowding. This represents an innovative and relevant indication for the future improvement of insect rearing from a welfare perspective, especially at an industrial scale.

## 1. Introduction

According to the World Organisation for Animal Health, “animal welfare refers to the physical and mental state of an animal in relation to the conditions in which it lives and dies. An animal experiences good welfare if it’s healthy, comfortable, well nourished, safe, not suffering from unpleasant states such as pain, fear, and distress, and if it is able to express behaviors that are essential for its physical and mental state ” [1].

Since May 1993, the Farm Animal Welfare Council, in its report on priorities for animal welfare research and development, has pointed out the need for advancing research and development on animal welfare as a topic of public concern [2].

It is fundamental to recognize the diversity and complexity of life, designing research for understanding living systems by creating a minimum of harm or discomfort to living organisms [3,4]. Moreover, Cohen [3,4] underlined the relevance of inflicting the minimum of pain, trauma, or damage to the subjects under study, understating if the experiment is necessary for obtaining the required information. The primary focus is on handling subjects, specifically insects, to ensure their well-being and health while minimizing stress [3,4].

The idea of “comfort”, in the meaning of how we manage insects in our rearing practice, should be considered in order to obtain the best conditions and to avoid adverse stimuli. As a priority, we should maintain the same conditions pertaining to a wild population of insects in an artificial environment as the rearing method adopted in the current insect breeding [3,4]. Therefore, the ideal conditions for insects in a rearing system should mirror those of wild insects, fostering an optimal environment that promotes the highest possible life quality in our insect rearing [3,4].

The assessment of animal welfare can be conducted considering the Five Freedom framework, first described in the “Brambell report”, which explained the minimum conditions that should be met in intensive farming [5]. These principles were formulated by F. Rogers Brambell in 1965 and include the following: (I) freedom from thirst, hunger, and malnutrition; (II) freedom from discomfort; (III) freedom from pain, injury, and disease; (IV) freedom to express normal behavior; and (V) freedom from fear and distress [2,6]. Although several, more modern, approaches to animal welfare emerged in the following decades, the Five Freedoms remain a crucial first step for assessing that the basic needs of farmed animals are met. The achievements obtained during the last decades in terms of animal welfare have been substantial, but unfortunately did not include the insect rearing sector, despite the growing interest in this topic [7]. Moreover, insects reared at the industrial level for the production of feed ingredients are considered as “farmed animals” by the law, underlining even more the lack of regulation on insects’ welfare at a European level [8].

Currently, there is a lack of evidence on insects’ sentience, but this does not equate to evidence of absence [9]. Numerous challenges and limitations exist in studying insect sentience, as the scientific tools available are still limited. Therefore, it is essential to apply the precautionary principle and treat insects as sentient beings until proven otherwise [7,9]. In particular, as reported by Cohen [3,4], the quality of our experiments can be maintained only if we can conduct them using the best components and answer all our inquiries. Insects’ “comfort” should be evaluated during our experiment, considering it as one of the main components of our ethical approach. The improvement of the basic knowledge about insect rearing is necessary in order to reduce the risk of causing discomfort for reared insects [3,4].

Recently, the International Platform of Insects for Food & Feed (IPIFF) stated the importance of considering the Five Freedoms framework in developing welfare practices encompassing the insects’ production process [10]. The first freedom, “freedom from hunger, thirst, and malnutrition”, requires the provision of adequate feed both in terms of quantity and nutritional value, as well as access to water. Similarly, “freedom from discomfort” and “freedom to express normal behavior” need to be guaranteed in insect rearing mainly throughout the rearing process. Grandin [11] emphasized the importance of using numerical measurements to improve animal welfare and productivity in livestock species. Similarly, Barrett et al. [12] suggested using population-level mortality as a very coarse proxy for the subjective welfare of individual insects. Furthermore, they highlighted sublethal factors that may reduce BSF welfare in commercial settings, such as abiotic conditions (temperature, humidity/moisture, substrate aeration, light, pupation substrates, and adult spatial needs) and stressed the importance of evaluating how these factors impact welfare beyond individual mortality.

Performance indicators are currently used to study how various factors affect insect rearing. Their application as indirect welfare indicators represent progress in the field, furthering the knowledge in this area.

Insects are ectotherms, and numerous environmental factors influence their behaviors, including feeding, growth, locomotion, mating, immune function, and sensory input [13,14]. More in detail, each insect species requires a specific evaluation, and this paper focuses on the *Hermetia illucens* L. (black soldier fly, BSF).

Due to its polyphagous nature, the BSF feeds exclusively during its larval stage [15] and can use different rearing substrates [16,17,18,19,20,21,22,23,24]. In BSFL, larval protein, crude fat content and development time are significantly influenced by the nutrient concentration in the substrate (both the quality and quantity of the diet) and larval density [25,26].

Firstly, the feeding rate refers to the quality and quantity of feed that meets the nutritional needs of the insect. A single BSF larva can consume between 25 and 500 mg of substrate per day, depending on various factors such as particle size, fiber content, larval size and age, and moisture content [27]. Diener et al. [28] found that 100 mg/larva/day of chicken feed ensured larval growth and high organic matter degradation. Similarly, Nyakeri et al. [29] compared larval growth at different feeding rates (100, 150, 200 and 250 mg/larva/day) and diets (food waste, brewer’s waste, and fecal sludge), concluding that higher feeding rates significantly improved larval growth and performance with optimal results at 200 mg/larva/day of substrate. Similarly, Abduh et al. [24] tested feeding rates of 50, 75, and 100 mg of feed/larva/day, rearing BSF on a diet formulated with 0–20% of seed cake Philippine tung (*Reutealis trisperma*) content. Larvae grew faster with higher feeding rates (100 mg/larva/day), also registering higher productivity, probably due to increased protein availability. On the contrary, when food is scarce, development time and mortality increase, while adult body size decreases [30]. 

Environmental conditions play a crucial role in BSF feeding rates; for example, temperatures below 21 °C reduce feed intake and, consequently, the larvae’s ability to process waste [31]. Similarly, the house fly (*Musca Domestica* L., order Diptera) showed reduced feeding rate and locomotor activity when fed the bacterium *Brevibacillus laterosporus* (Bacillales: Paenibacillaceae) [32]. As suggested by Kortsmit et al. [33], overcrowded conditions can lead to thermal stress, reduced feeding and growth, resulting in delayed developmental and reduced final weight [34]. Parra Paz et al. [35] studied different feeding rates by using a Control diet (laying hen feed), identifying a feed intake limit beyond which the larvae would not consume. In addition, feeding frequency plays a significant role in the growth of BSF. In batch feeding, feed is administered only once at the beginning of the experiment, while in incremental feeding, feed is administered every other day of the experiment [36]. In batch feeding, microbial activity was found to lead to spoilage and reduce nutrient availability; additionally, larvae showed a preference for fresh substrate [37,38]. In contrast, moments of nutrient starvation can occur in a continuous system [21]. Banks et al. [17] found that batch feeding results in higher weight gain and longer prepupal development time compared to incremental feeding, while Meneguz et al. [20] reported the opposite trend.

Secondly, as underlined by Barragan-Fonseca et al. [25], Diener et al. [28] and Tomberlin et al. [39], larval density affects the rate of BSF development. Larval density significantly affects the bioconversion of organic matter into BSF body mass. Abduh et al. [24], applying 1–1.4 larvae/cm^2^ as larval density, registered lower productivity compared to other research with higher larval densities (5 larvae/cm^2^).

While larvae naturally tend to aggregate, overcrowded conditions slow larval development due to competition for feed [40]. Additionally, larval aggregation increases the substrate temperature, which can have positive effects in some insect species, such as improving feed assimilation [41] or protecting them from low environmental temperatures [40]. The effects of density on heat dissipation and water evaporation from the substrate can also alter substrate properties, consequently affecting larval growth. For example, high densities can degrade substrate quality due to the accumulation of larval waste products [42]. However, some studies have shown that high larval densities are associated with increased bacterial densities, which may enhance larvae’s access to bacteria-recycled nutrients, resulting in more efficient nutrient absorption [43]. Conversely, at low densities, larvae may not achieve sufficient substrate conditioning and cooperative digestion [44,45].

Since large-scale BSF rearing is still in its early stages, determining the importance of feed quality and quantity from a welfare perspective is crucial. Similarly, rearing density represents a key parameter for BSF welfare, as larval density significantly affects the conversion of organic waste [17,46,47].

Given the increasing need to ensure good welfare parameters in sustainable BSF farming, this article aims to evaluate the effects of different feeding rates and rearing densities on two individual substrates. Performance parameters were considered as indirect indicators of larvae welfare. As suggested by Cohen [3,4], we should consider all these thoughts to limit damage to insects during their rearing process. Furthermore, this approach helps us evaluate and identify useful parameters to monitor the insects’ well-being during research.

## 2. Materials and Methods

### 2.1. Colony Status

The BSF colony maintained in the laboratory of BEF Biosystems (Casalnoceto, AL, Italy) was used for this study. BSF adults were housed in a steel-frame cage (100 cm × 63 cm × 110 cm) with a mosquito net cover and equipped with LED lighting suitable for BSF, with a 12:12 light:dark cycle. They were kept in a climate-controlled room at 27 ± 1 °C and 65 ± 5% relative humidity. Water was always available. Wooden sticks were used as oviposition substrates, checked and replaced every second day. Eggs were collected with a paintbrush, transferred to plastic cups [43] and moved to plastic boxes (60 cm × 40 cm × 12 cm). After hatching, larvae were fed a mixture of chicken feed and water until the test began, to reduce the external factors that could affect the neonatal larvae development.

### 2.2. Waste Ingredients and Dietary Substrates

Diet ingredients included vegetable by-products (Stroppiana, Turin, Italy), meat ingredients (Barf, Tortona, Italy) and commercial hen feed (Progeo, Reggio Emilia, Italy).

Two diets were formulated as follows:

- Omnivorous (O): 16.66% potato, 16.67% carrot, 16.67% thresher of beer, 25.00% epiglottis of beef, 25.00% cod.
- Control (C): 72.72% water, 27.28% laying hens feed.

The chemical composition of the diets is reported in Table 1, as determined in Belperio et al. [48].

The vegetable and meat materials were prepared by carefully mixing and reducing the material to a size below <3 mm using a mixer (Sirman, Katana 20VV, Curtarolo, PD, Italy).

### 2.3. Experimental Design

A homogeneous pool (2 kg) of 6-day-old BSF larvae was separated from their rearing substrate using a vibrating sieve (2 mm, VibroWest MR 24//5.5.5, Milan, Italy). The individual weight of a representative sample of 300 larvae was measured using a digital scale (U.S. Solid, USS-DBS46-1, Cleveland, OH, USA). The 300-larvae sample consisted of three different sampling units (100 larvae each), taken from different areas of the rearing box. The average larval weight was 25.62 ± 9.45 mg in trial 1 (feeding rate trial), while 28.85 ± 2.13 mg in trial 2 (density trial).

Due to the large experimental design, the larval mass representing the number of larvae needed for each replicate was calculated starting with the individual larval weight.

The 6-day-old larvae were reared in plastic boxes (32 cm × 23.5 cm × 11.5 cm; taking into account the curvature of the surfaces, the internal measurements of the base was 29 cm × 21 cm), with 4 replicates per treatment. The boxes were placed on a table in randomized order and let at constant temperature and humidity (27 ± 1 °C, 65 ± 5% relative humidity). A ventilator was in the room (airflow 1.8 m/s) to remove excess humidity from day 2 onwards. The lids of the boxes were kept until day 4 (to avoid flies contamination), then removed for the rest of the trial.

#### 2.3.1. Trial 1—Feeding Rate

Different feeding rates on each substrate (Control and Omnivorous) were tested:

- 50 mg of feed/larva/day (C.50, O.50);
- 100 mg of feed/larva/day (C.100, O.100);
- 200 mg of feed/larva/day (C.200, O.200).

Each box was filled with 2000 estimated larvae, for evaluating the rearing performances. Considering day 0 as the moment of larvae sowing, the duration of the trial was fixed to day 0 + 8 days, with the end of the trial corresponding to an adequate dryness on the material suitable for industrial sieving. The feed was administered all together on day 0.

#### 2.3.2. Trial 2—Density

Omnivorous and Control diets were tested at three different rearing densities:

- 5 larvae/cm^2^ (C.5, O.5, 3045 estimated larvae);
- 10 larvae/cm^2^ (C.10, O.10, 6090 estimated larvae);
- 15 larvae/cm^2^ (C.15, O.15, 9135 estimated larvae).

Considering day 0 the moment of larvae sowing, the duration of the trial was fixed to day 0 + 8 days, with the end of the trial corresponding to an adequate dryness on the material suitable for industrial sieving. The feeding rate was planned to be 100 mg feed/larva/day. Due to the high amount of substrate required and to promote optimal BSF feed intake, the diet administration was divided into 3 batches for each treatment, administered on days 0, 3, and 6.

Applying the same experimental design and environmental conditions, a specific setup was organized for both trial 1 (feeding rate) and trial 2 (rearing density) to precisely evaluate the survival rate (SuR) with a countable amount of larvae. The SuR trials were carried out in boxes (8 cm × 8 cm × 5 cm), with 100 larvae (manually counted) for each replicate in trial 1 (feeding rate), while in trial 2 (rearing density) 320, 640 and 960 larvae (manually counted) were used, respectively, for each replicate of density (5, 10, 15 larvae/cm^2^).

### 2.4. Growth Performance of Black Soldier Fly Larvae

At the end of both trials, each box was weighed using a Kern scale (GAB 12K0.1N, KERN, Balingen, Germany, maximum 6 kg, d = 0.05 g)) For each replicate, larvae and frass biomasses were carefully separated using a vibrating sieve (VibroWest MR 24//5.5.5, Milano, Italy) and weighed (Kern, GAB 12K0.1N). Growth rate (GR; Formula (1)) and feed conversion ratio (FCR; Formula (2)) were calculated to assess the speed at which an insect increases in weight and the insect’s efficiency in converting feed into body mass.

At the end of the experiment, a representative sample of larvae (100) was weighed individually using a digital scale (USS-DBS46-1, U.S. Solid, Cleveland, OH, USA) to determine the average larval weight. In addition, a subsample of 10 larvae was frozen and their length measured using ImageJ software (Version 1.53, 2022). The feeding rate (FR, amount of feed that an insect consumes within a specific time period) was calculated as indicated in Formula (3). Substrate reduction (SR), intended as the decrease in the mass or volume of a substrate due to insect feeding and digestion, was indicated in Formula (4). Moreover, waste reduction index (WRI, a measure of an insect’s ability to reduce or break down organic waste) is presented in Formula (5). Efficiency of conversion of digested food (ECD, intended as the proportion of digested food that an insect converts into its own body mass, Formula (6)) was evaluated.

Lastly, the survival rate (SuR, the percentage of insects that survive over a specific period), was assessed by counting the larvae of the specific small-scale trial for each treatment (see material and methods, Section 2.3.2) and applying Formula (7). The formula used are available in Table 2.

### 2.5. Chemical Composition of Black Soldier Fly Larvae

Samples of larvae were analyzed at the Animal Production and Food Safety Service (SPASA) laboratories of the Department of Veterinary Medical Sciences (DIMEVET) of the University of Bologna, Italy. Samples were stored at −20 °C to induce larval death and to maintain their chemical properties. Larvae and substrates were then freeze-dried at −40 °C for four days using (Olsa, Milano, Italy) to remove all moisture in the organic matter.

All samples were weighed using a Kern GAB balance (GAB 12K0.1N, KERN, Balingen, Germany, maximum 6 kg, d = 0.05 g) then shredded with a blender (Broyeur mélangeur MB 950G KINEMATICA, Malters, Switzerland) to reduce the particle size. The shredded material was stored in hermetically sealed plastic containers to avoid external contamination or moisture accumulation. AOAC International [51] procedures were used on BSF larvae samples to determine DM (method no. 930.15), ash (method no. 942.05), CP (method no. 976.06), ether extract (method no. 920. 39) and starch (method no. 996.11). The CP content of BSF larvae was calculated using the nitrogen-to-protein conversion factor of 4.76 as suggested by Janssen et al. [52]. Neutral detergent fiber (NDF) was determined by refluxing 0.5 g of sample for 1 h at boiling temperature in a medium-porosity crucible, using a Fibertec 2010 system (Foss Tecator, Foss Italia srl, Padova, Italy) [53]. A commercially available neutral detergent solution was used, containing deionized water, sodium lauryl sulfate, disodium EDTA, sodium borate, disodium phosphate, ethoxyethanol and anhydrous sodium sulfite. Residual nitrogen (ADF) and acid detergent lignin (ADL) were analyzed according to Van Soest et al. [53].

The proximate composition of BSF larvae was expressed as % on a dry matter basis (DM). All analyses were performed in duplicate.

### 2.6. pH Evaluation

In trial 2, the pH of the substrates was registered using a pH meter (PHH-7011, Omega, Biel, Switzerland) starting from day 0 before larvae sowing. The pH meter was equipped with a temperature compensation probe. The measurement was taken daily at the same time. pH values were not recorded on re-feeding days, since they would be affected by the freshly added diet and not yet colonized by larvae.

### 2.7. Statistical Analysis

The statistical analysis of data was performed using R software (version R 4.1.2 2023) with the interface R-studio (version 2023.03, 2023). The effects of diet, feeding rate (trial 1) or density (trial 2), and their interactions on performance (response variables) were assessed using Linear Mixed Effects models, applying the nlme package [54]. Diet and feeding rate (trial 1) or density (trial 2) were treated as fixed effects (covariates), while the rearing boxes were considered as a random effect. In addition, a multivariate comparison of the covariates was conducted in a post hoc analysis using the emmeans package [55]. Sets of means estimated for each group of the covariate of interest were compared using the Mahalanobis distance and Hotelling’s T statistic [56]. The root mean squared error (RMSE) was calculated to evaluate the model accuracy. Significance was declared at *p* ≤ 0.05. For pH statistical analysis, one-way ANOVA was applied, which indicated a significant effect of the treatment on the variable with a *p* < 0.05. Tukey’s HSD test followed for the multiple comparisons of means at a 95% family-wise confidence level (significant with *p* < 0.05). An appendix reporting the mean values for each parameter obtained for a specific diet and treatment is available as supplementary material (Tables S1 and S2).

### 2.8. Process Modeling and Optimization

Using Design Expert software (Stat-Ease, Inc., Minneapolis, MN, USA, version 11.1.2.0), the optimization of rearing parameters for both Control and Omnivorous diets was investigated. Each dietary regime was analyzed separately, using a Central Composite Design (CCD) that considered feeding rate (three different levels: 50, 100, and 200 mg feed/larva/day for trial 1) and density (three different levels: 5, 10, 15 larvae/cm^2^ for trial 2). The response factors included larval weight, growth rate, waste reduction index, efficiency of conversion of digested food, substrate reduction, and feed conversion ratio. The optimization goal was to maximize all factors except FCR, which should be minimized. The significance level of 3 was chosen for each factor.

## 3. Results

### 3.1. Growth Performance and Waste Reduction Efficiency of Black Soldier Fly Larvae

#### 3.1.1. Trial 1—Feeding Rate

It is relevant to highlight that in treatments C.50 and C.100, 200 g and 50 g of water were added, respectively, due to excessively fast substrate drying. Furthermore, the experiment concluded one day early (on day 7) for treatments C.50, C.100, O.50, and O.100, as they had already reached the optimal level of dryness of the boxes assessed visually. On the contrary, boxes C.200 and O.200 completed the trial on day 12 instead of day 8. Consequently, the actual feeding rates (feed administered/larva/days of trial) were slightly different than the planned values, due to the different duration of the trial. The trial was therefore conducted with feeding rates of 60, 114 and 134 mg feed/larva/day. The development of BSF larvae reared on different substrates with different feeding rates is shown in Table 3. The results showed a significant difference in final larval weight between the feeding rates and diets (*p* < 0.001). Similar trends were noted in final larval biomass, with higher results in the Omnivorous diet and at a feeding rate of 200 mg/larva/day. The GR was improved with the Omnivorous diet and the highest feeding rate. Notably, the FCR showed lower values in the Omnivorous diet and at the feeding rate of 50–100 mg feed/larva/day (*p* < 0.001). No significant effect of the diet was observed on larval length (*p* = 0.38). The WRI and ECD were higher for the Omnivorous diet and at the feeding rate of 50 m feed/larva/day. Interestingly, a significant diet (D) × feeding rate (FR) interaction was observed for final larval biomass, SR, WRI and larval length (*p* < 0.001).

Interestingly, the survival rate was not affected by the dietary regimes, feeding rate, or their interaction.

#### 3.1.2. Trial 2—Rearing Density

The rearing performance of BSF at different densities is presented in Table 4.

A significant difference (*p* = 0.016) was observed in final larval weight between the treatments at densities of 10 and 15 larvae/cm^2^, with a lower final larval weight recorded at the highest density. The final larval biomass of the C diet was significantly higher than that of the O diet (*p* < 0.001).

No significant differences in frass biomass were found between the densities of 10 and 15 larvae/cm^2^, while a lower biomass was obtained at a density of 5 larvae/cm^2^.

Notably, improved performance in terms of substrate reduction and FCR was observed for the Omnivorous diet (*p* < 0.001) at densities of 10 and 15 larvae/cm^2^ (*p* = 0.021 and 0.011, respectively) compared to 5 larvae/cm^2^. The ECD index did not show significant differences among the densities (*p* = 0.771), while the Control diet achieved significantly higher results than the Omnivorous diet (*p* < 0.001).

A comparison between the theoretical and actual feeding rates revealed interesting data. For both dietary regimes, the real daily feeding rate increased at low-density rearing due to the rapid drying of the substrate in the boxes, leading to an early conclusion of the trial. Conversely, at density of 15 larvae/cm^2^, the trial extended beyond 8 days, resulting in a lower daily feeding rate. The survival rate registered significative differences depending on the dietary regimes (*p* = 0.024), with improved results in the Omnivorous diet compared with the Control diet.

### 3.2. Chemical Composition of Black Soldier Fly Larvae

#### 3.2.1. Trial 1—Feeding Rate

Dry matter content was affected by dietary regime (*p* = 0.051), feeding rate, and their interaction (*p* < 0.001) (Table 5).

Crude protein content was higher in larvae fed the Omnivorous diet at a feeding rate of 50 mg/larva/day. Interestingly, crude fat followed the same trend for both dietary regimes, with significantly higher fat accumulation in larvae fed at a rate of 200 mg/larva/day (*p* < 0.001). No significant differences were observed in ash content between feeding rates of 50 and 100 mg/larva/day, both of which showed higher ash content than the other treatments (*p* = 0.001).

#### 3.2.2. Trial 2—Rearing Density

Table 6 shows the chemical composition of larvae at the end of the rearing period. Dry matter content showed significant differences between the densities of 5 and 10 larvae/cm^2^ (*p* = 0.006). Crude protein content revealed an interesting trend, with higher values recorded for the Omnivorous diet at the lowest rearing density (*p* < 0.001). However, no significant differences in protein content were observed between the densities of 10 and 15 larvae/cm^2^. Interestingly, for the protein content, a significative interaction effect of the diet with the density was registered (*p* = 0.016). Significant differences (*p* < 0.001) in fat accumulation were observed when comparing the Control and the Omnivorous diet, as well as among the density treatments, with the highest value observed in the Omnivorous diet at a density of 10 larvae/cm^2^. As expected, the greatest ash content was recorded in the Control diet at 10 and 15 larvae/cm^2^ densities.

### 3.3. Process Modeling and Optimization

#### 3.3.1. Trial 1—Feeding Rate

**Figure 1:**
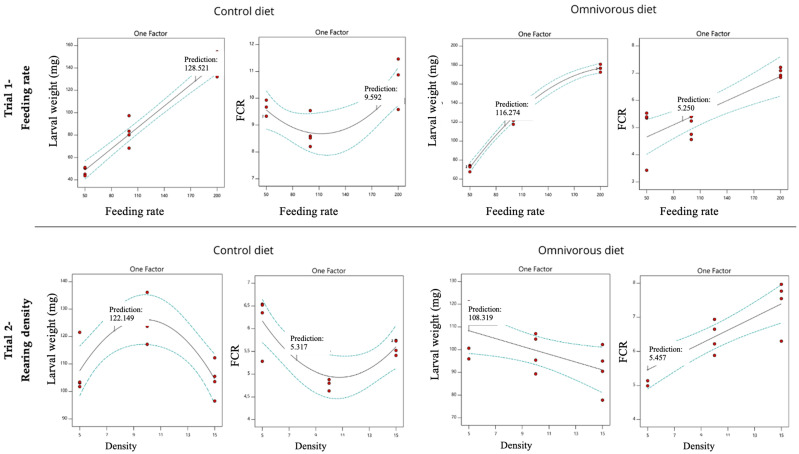
Larval weight and feed conversion ratio (FCR) optimization for Control and Omnivorous diets at different feeding rates (trial 1) and different rearing densities (trial 2). Dots (red): observed experimental data; dashed line (green): 95% confidence intervals; solid line (black): predictive model; label “Prediction”: optimal predicted value.

The optimal performance for the Control diet was achieved at a feeding rate of 175 mg feed/larva/day, while for the Omnivorous diet, the optimal performance was obtained at a lower feeding rate (90.2 mg feed/larva/day) (Table 7, Figure 1). In fact, the process modeling software highlighted that the above-mentioned feeding rate (for Control and Omnivorous diets) lead to the optimal rearing process output by considering larval weight, grow rate, substrate reduction, waste reduction index, efficiency of conversion of digested food and feed conversion ratio.

#### 3.3.2. Trial 2—Rearing Density

In the Control diet, the optimal performance occurred at a density of 7.57 larvae/cm^2^, while lower densities in the Omnivorous diet led to improved performances. Specifically, a density of 5.00 larvae/cm^2^ was identified as the optimal one (Table 7, Figure 1). In fact, the process modeling software highlighted that the above-mentioned rearing density (for Control and Omnivorous diets) leads to the optimal rearing process output by considering larval weight, grow rate, substrate reduction, waste reduction index, efficiency of conversion of digested food and feed conversion ratio.

### 3.4. pH Variation

**Figure 2:**
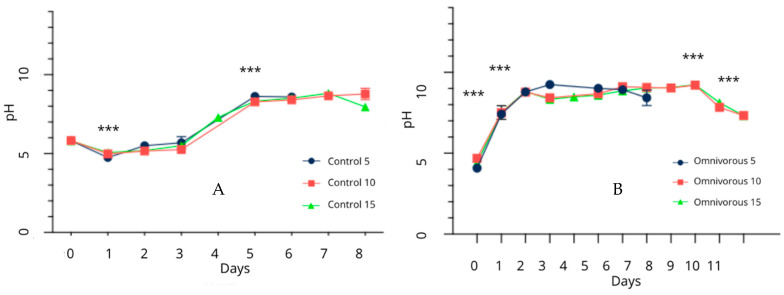
pH variation during the trial in the Control (**A**) and Omnivorous (**B**) treatments. Asterisks indicate significant differences between one day and the previous pH reading available (***, *p* < 0.001).

Figure 2 shows the pH variation in the Control and Omnivorous diets during trial 2, where different rearing densities (5, 10, and 15 larvae/cm^2^) were tested.

Initially, the Control diet exhibited a pH of 6 (i.e., slightly acidic) at day 0. After 24h, a significant decrease in pH was observed (*p* < 0.001), indicating increasing acidification until day 3. No measurements were taken at day 4 (re-feeding day), but by day 5, a significant shift towards alkalinity was noted (*p* < 0.001). The pH then remained stable until the end of the trial (day 8). For the Omnivorous diet (Figure 2), the initial pH was acidic (pH 4 at day 0). With the sowing of the larvae and the beginning of the trial, the pH reached neutral levels by day 1, showing a significant difference from day 0 (*p* < 0.001). This basification process continued through day 2 (*p* < 0.001). From day 3 to day 8, pH values remained relatively stable across all Omnivorous treatments. For O.10 and O.15 groups, the pH started to decrease after day 9, presenting values near to the neutrality. A significant difference was observed on day 10 and 11, with a final pH drop to neutral values.

## 4. Discussion

Building on the optimal growing substrate identified in previous trials [48,57], this study aimed to investigate the effects of different feeding rates and rearing densities on the growth of black soldier fly larvae from a welfare perspective. The aim is to evaluate the “comfort” of insects as reported by Cohen [3,4], which has been considered indirectly by placing the BSFL in the best rearing conditions. There are no direct parameters, until now, that we can use to evaluate the comfort state of our insects. As suggested by Cohen [3,4], we need to limit the damage caused to insects by evaluating how the vital parameters are influenced during the rearing.

Feeding rate and density are two rearing parameters that are strongly correlated in BSF processing. Although the literature on this topic is still limited, understanding these factors is crucial to optimize productivity and efficiency in rearing practices.

### 4.1. Trial 1—Feeding Rate

#### 4.1.1. Feed Intake, Rearing Performances and Larvae Chemical Composition

In our trial, it was not possible to reach the high feeding rate planned, equal to 200 mg feed/larva/ day; in fact, the maximum feeding rate obtained was 134 mg feed/larva/day. With 50–100 mg feed/larva/day, the amount of substrate was suitable to be consumed quickly by the larvae. On the contrary, the dose of 200, exceeding the daily intake capacity of the larvae, required more time for consumption, extending the duration of the trial for obtaining the suitable dryness of the residual substrate.

As suggested by Parra Paz et al. [35], BSF larvae have an upper limit of feed intake that cannot be exceeded, which can be considered as a threshold for ensuring “Freedom from discomfort”. This was also visible in the pH reduction (acidification) that occurred in this trial. Digestive enzymes, such as amylases, lipases, and protease (trypsin and chymotrypsin), play a crucial role in BSF digestion process [58,59,60]. These enzymes are influenced by factors like diet composition [61], gut environment, pH [20], and ambient temperature [62]. During the rearing, substrate temperatures can rise to 45 °C, the optimal temperature for proteolytic activity (around pH 8), enhancing the efficiency of BSF larvae in bioconversion [59,62].

The Control diet resulted in lower growth performance compared to the Omnivorous diet at both feeding rates of 100 and 200 mg feed/larva/day, suggesting that the Control substrate is nutritionally less adequate than the Omnivorous diet, composed of fruit and vegetable by-products and meat. This aligns with Cattaneo et al. [57] and Addeo et al. [63] who observed that the nutrient richness and variety of an Omnivorous diet supplied BSF with all the nutrients needed to grow efficiently.

Optimal larval growth rate (11.16 mg/day) and the most favorable (i.e., the lowest) FCR (6.85) were achieved with a planned feeding rate of 100 mg feed/larva/day under the Omnivorous diet, in agreement with Diener et al. [28]. Our results were similar to the outcomes of Abduh et al. [24], who detected a feeding rate of 100 mg/larva/day as the optimal setting for improving rearing productivity.

Process optimization analysis identified an optimal feeding rate of 90 mg feed/larvae/day for the Omnivorous diet and 175 mg feed/larvae/day for the Control diet. These results are in agreement with Parra Paz et al. [35], who found that feeding rates between 95 and 163 mg feed/larva/day yielded improved growth performances in BSF larvae reared for biomass production. Interestingly, a marked dehydration was visually observed in some of the treatments under analysis. In fact, Diener et al. [28] explained that, when applying a restricted food ration, an excessively high surface-to-volume ratio may accelerate the drying process. As a result, drier feed is converted less efficiently by BSF larvae compared to substrates with higher moisture content, potentially compromising larval welfare by violating the freedom from discomfort and hunger. To mitigate this effect and avoid negatively influencing the trial, adjustments were made to the trial duration, which resulted in slight changes in the actual feeding rates compared to the planned ones. On the other hand, treatments fed at 200 mg/larva/day required longer than planned to complete the trial due to the larger substrate mass and the consequently extended drying time, leading to an actual feed intake of 134 mg/larva/day for both the Omnivorous and the Control diet.

#### 4.1.2. Welfare Perspective on Feeding Rate

Growth rate, substrate reduction and FCR are key parameters that can be used as indicators of overall insect welfare, similar to what Grandin [11] suggested for livestock species, underlining the importance of using numerical measurements to improve both animal welfare and productivity.

An adequate quantity and quality of food allow larvae to satisfy their needs, therefore addressing the first freedom (freedom from hunger and thirst) and as explained by Cohen [3,4]. The “comfort” of the larvae is visible by a satisfying final weight, directly related to the feed intake and then to the possibility of accumulate adequate amount of fat reserves for the adult stage, that will be used for reproduction. On the contrary, excessive feeding rates can violate both the second and fourth freedoms (freedom from discomfort and freedom to express normal behavior), since the larvae are not able to move freely in the substrate nor able to colonize it as preferred. From a welfare perspective, in addition to techniques that optimize substrate processing by larvae (i.e., modifying the texture, microbial levels, pH, moisture, and nutrient composition), it is crucial for producers to precisely manage the quantity of feed provided to the larvae [12].

Overfeeding can create anaerobic conditions, reduce the pH to acidic levels [35], and encourage pathogen and bacteria proliferation. In this regard, Tomberlin et al. [64] recommends introducing additional feed only when no more than 30–50% of the substrate remains undigested.

Moreover, overfeeding can lead to heavier larvae, higher metabolic rates, and increased risk of overheating [35]. Limited knowledge is available regarding individual pain, fear, discomfort, or distress in insects, particularly in BSF larvae. For this reason, Barrett et al. [12] proposed using population-level mortality as a welfare indicator.

Mortality is an observable sign of stress, and if BSF are sentient, it is related to negative subjective states. Furthermore, sublethal factors such as diseases/parasites, unfavorable abiotic conditions (temperature, humidity/moisture, substrate aeration, light), adult and larval nutritional needs, injury and crowding may also reduce BSF welfare.

Larvae nutrition and specific rearing parameters can help address three of the Five Freedoms—(I) freedom from hunger and thirst, (II) freedom from discomfort, and (IV) freedom to express normal behavior—thereby improving BSF welfare. Performances’ outcomes can be interpreted as a rapid, straightforward, and non-invasive indicator.

This research, merging performance parameters and process modeling, showed that with the Omnivorous diet, real feeding rates around 90 mg feed/larva/day support an efficient larval growth while maximizing substrate utilization. From a welfare perspective, this feeding regimen fulfills the first, second, and fourth freedoms, as detailed above, and as reported by Cohen [3,4], it increases the “comfort” of larvae and reduces the suboptimal conditions.

### 4.2. Trial 2—Rearing Density

#### 4.2.1. Rearing Densities

Our study confirmed that rearing density plays a pivotal role in optimizing BSF larvae growth, with significant differences observed between the Control and Omnivorous diets. Specifically, for the Control diet, process modeling and optimization underlined optimal performance at density of 7.57 larvae/cm^2^, while for the Omnivorous diet, density of 5.00 larvae/cm^2^ led to the best results. Interestingly, the survival rate did not register significant differences across density treatments, being only influenced by the diet.

These findings highlight the importance of considering both diet composition and rearing density in optimizing larval growth and overall rearing efficiency, in line with those suggested by Diener et al. [28] and Parra Paz et al. [35] who recommended a density of 5 larvae/cm^2^. In accordance with this guideline, Abduh et al. [24] registered lower larval performances when applying rearing densities of 1–1.4 larvae/cm^2^.

Our results for the Omnivorous diet at 5 larvae/cm^2^ support this conclusion, as this density facilitated the most efficient growth and substrate utilization. Jones et al. [65] found that lower densities (500 larvae/4 L boxes) led to faster development and improved survival rates compared to higher densities.

In terms of the Omnivorous diet, our results support this information. The higher optimal density observed for the Control diet suggests that dietary composition significantly influences the interaction between density and larval performance.

Moreover, in addition to rearing density, larvae tend to aggregate, increasing the substrate temperature. This phenomenon can, in turn, enhance feed assimilation [41] or negatively change feed features such as temperature, aeration etc. [42].

On one hand, high larval densities are associated with higher bacterial densities, which may improve the nutrient absorption by BSF [43]. On the other hand, at low densities, larvae may not achieve sufficient substrate conditioning and cooperative digestion [44,45]. This is reflected in our data: in the Control diet at low densities (5 larvae/cm^2^), the larvae did not exhibit efficient growth, whereas they performed well on the Omnivorous diet due to the nutrient richness of this substrate. However, despite the benefits of higher bacterial densities and enhanced nutrient absorption in the Omnivorous diet, the larvae experienced limited movement within the boxes, which did not represent an optimal condition. This information is particularly relevant for industrial production, where even small deviations in larval density can have substantial impacts on yield [65], but numerous factors can efficiently be controlled and tailored.

#### 4.2.2. Change in pH of the Substrate During the Rearing

BSF can survive at a wide range of pH, from 2 to 11 [12]. However, pH values under 4 do not ensure optimal growth performance [20,66,67]. Initially, the pH of the substrate shifts from neutral to acidic [68], due to microbial products and excretions from the larvae such as short-chain fatty acids, ammonia, and uric acid [69,70].

After approximately 10 days of rearing, a final basic pH (between 8.5 and 9.5) can be achieved [20,67]. This alkalization is attributed to the production of ammonium ions (NH +) and ammonia [66,69,71]. Green et al. [42] also observed that BSF can neutralize the acidity of compost leachate.

In the current study, the Omnivorous treatments exhibited a stable pH trend from day 0 to day 2, with an initial pH of 4. As the trial progressed, differences among densities emerged. The treatment Omnivorous 5 larvae/cm^2^ maintained a stable pH from day 2 to 6 but showed a decrease on day 7 (the last day of the trial), with a rapid alkalization of the substrate. Treatments Omnivorous 10 larvae/cm^2^ and Omnivorous 15 larvae/cm^2^ displayed a similar trend, with stable pH levels until day 9, followed by a decrease in the final two days of the trial. Both treatments ultimately achieved a neutral pH.

#### 4.2.3. Relationship Between Density, Feeding Rate and pH

While larvae can stabilize pH, excessive feeding may lead to anaerobic conditions due to undigested feed, resulting in a decrease in pH that negatively affects larvae growth [35]. As an indicator of feeding behavior, pH suggests how larvae at density of especially 15 larvae/cm^2^ require more time to degrade the material. The most favorable pH values (close to neutrality) were obtained at lower density and feeding rate [35]. Therefore, the present study concluded that a density of 5 larvae/cm^2^ enhances larvae rearing on rich substrates like the Omnivorous diet.

#### 4.2.4. Welfare Perspective on Density

High density can inhibit larvae movement within the substate, thereby violating the second and fourth freedoms, specifically freedom from discomfort and freedom to express normal behavior. This explains why the planned feeding rate was not achieved, especially in high-density treatment (15 larvae/cm^2^) where uneaten food remained in the boxes at the time of re-feeding, making it spatially impossible to add more food. Consequently, the larvae food consumption capacity was limited to what was available in their immediate vicinity, also risking violations of the first freedom (freedom from hunger, thirst, malnutrition). From a welfare perspective regarding pH evaluation, larvae tended to achieve larger sizes in neutral to basic rearing substrates [66,67]. However, as Barrett et al. [12] noted, size is not necessarily a reliable welfare indicator. On the contrary, the rearing density can be considered one of the most important parameters to provide the best “comfort” we need for our larvae, as reported by Cohen [3,4], in order to reduce the inquiries and optimize our experimental conditions. The pH trends observed in the Omnivorous diet treatment better aligned with optimal larvae growing conditions.

### 4.3. Limitations of the Study

In the feeding rate trial, no management difficulties emerged, except for a natural difference in the times of feed consumption between treatments and drying of the materials, compatible with the process in progress.

On the contrary, in the density trial, the height of the boxes emerged as a limitation, as space constraints prevented the addition of all the planned feed at higher densities.

It is possible that maintaining the same surface area while increasing the height of the containers for higher densities could create a more favorable food stratification, allowing the larvae to move effectively and degrade the material [20]. Optimizing the shape and geometry of the containers is crucial from an industrial perspective, as these factors directly affect production performance.

The lack of direct parameters that can evaluate the “comfort” of our reared insect, as suggested by Cohen [3,4], should be evaluated in future studies. This would optimize the overall rearing, avoiding suboptimal conditions and increasing our level of knowledge of insects as sentient animals, guiding the creation of useful rearing standards.

## 5. Conclusions

With the aim of improving the welfare of BSF larvae, the present study investigated how different feeding rates and densities affect the performance and well-being of larvae during the rearing process. Control and Omnivorous substrates were analyzed, comparing feeding rates of 50, 100 and 200 mg feed/larva/day (trial 1), and densities of 5, 10 and 15 larvae/cm^2^ (trial 2). By applying the Five Freedoms framework in combination with process optimization modeling, we identified the Omnivorous diet with a feeding rate of approximately 90 mg feed/larva/day as the most effective solution for addressing the first freedom (freedom from thirst, hunger, malnutrition) and the second freedom (freedom from discomfort). At a high rearing density (15 larvae/cm^2^), the insects were unable to move freely within the substrate, thus violating both the second and fourth freedoms: freedom from discomfort and freedom to express normal behavior, respectively. In addition, in high rearing density (15 larvae/cm^2^), larvae did not consume all the administered feed. In terms of rearing performance (including final larval weight, growth rate, survival rate, and process optimization), a density of 5 larvae/cm^2^ was defined as optimal, particularly with the Omnivorous diet.

Future evaluations could focus on the temperature profiles of both the substrate and the environment, to determine if different temperature settings could enhance feeding intake. Additionally, investigating the impact of the container volumes on the rearing process could offer valuable insights into establishing optimal welfare conditions in industrial systems that require the management of large numbers of larvae. From an ethical standpoint, using the precautionary principle and avoiding any risk of causing discomfort in the reared insects should be the aim of our farming systems.

## Figures and Tables

**Table 1:** Proximate chemical composition of the dietary substrates used in the trials (Control and Omnivorous diets), expressed as % on dry matter (DM) basis, except where otherwise specified.

Chemical Composition	Control	Omnivorous
Dry matter (%)	27.2	25.6
Crude protein	14.4	31.8
Crude fat	5.35	13.4
Ash	13.9	6.77
Fiber	5.02	9.65
Neutral detergent fiber	17.3	33.2
Acid detergent fiber	8.23	13.9
Acid detergent lignin	2.73	4.90
Starch	33.8	20.3

**Table 2:** Formula used as performance indicator for BSF rearing. Unit of measurement and calculations are indicated (except where not applicable).

Formula Number	Title	Calculations	References
(1)	Growth rate—GR (mg/day)	$\frac{Average\;final\;larval\;weight\;\left(mg\right)-Average\;initial\;larval\;weight\;(mg)}{number\;of\;days\;of\;trial}$	[20,49]
(2)	Feed conversion ratio—FCR	$\frac{Initial\;feed\;biomass\;\left(g\right)-Final\;frass\;biomass\;(g)}{Final\;larval\;biomass\;\left(g\right)-Initial\;larval\;biomass\;(g)}$	Adapted by Ciptono et al. [50]
(3)	Feeding rate—FR (mg/day/larva)	$\left(\frac{Initial\;feed\;biomass\;\left(mg\right)}{days\;of\;trial}\right):estimated\;numer\;of\;larvae$	[28]
(4)	Substrate reduction—SR (%)	$\frac{Initial\;substrate\;weight\;\left(g\right)-final\;substrate\;weight\;\left(g\right)}{initial\;substrate\;weight\;\left(g\right)}\times 100$	[49]
(5)	Waste reduction index—WRI	$\frac{\frac{Initial\;substrate\;weight\;\left(g\right)-final\;substrate\;weight\;(g)}{initial\;substrate\;weight\;(g)}}{number\;of\;days\;of\;the\;trial\;(d)}\times 100$	[49]
(6)	Efficiency of conversion of digested food—ECD	$\frac{Final\;larval\;biomass\;weight\;(g)}{Initial\;substrate\;weight\;\left(g\right)-final\;substrate\;weight\;(g)}$	Adapted by Leong et al. [49]
(7)	Survival rate—SuR (%)	$100-(\frac{Initial\;number\;of\;larvae-final\;number\;of\;larvae}{Initial\;number\;of\;larvae}$ × 100)	n.a.

**Table 3:** Rearing performance of black soldier fly larvae reared depending on diet (D), feeding rate (FR) and their interaction (D × FR).

	Diet (D)		Feeding Rate (FR)			RMSE	*p*		
	C	O	50	100	200		D	FR	D × FR
Final larval weight (mg)	91.12 ^B^	124.64 ^A^	59.82 ^C^	103.75 ^B^	160.06 ^A^	5.09	<0.001	<0.001	0.034
Final larval biomass (g)	205.96 ^B^	311.32 ^A^	148.94 ^C^	257.31 ^B^	369.66 ^A^	10.7	<0.001	<0.001	<0.001
Final frass biomass (g)	359.27 ^A^	293.34 ^B^	134.08 ^C^	309.16 ^B^	535.65 ^A^	22.2	<0.001	<0.001	0.03
Growth rate (mg/day)	7.01 ^B^	11.15 ^A^	4.89 ^C^	11.16 ^B^	11.20 ^A^	0.65	<0.001	<0.001	0.002
Substrate reduction (%)	79.63 ^B^	85.16 ^A^	83.23 ^A^	80.68 ^C^	83.26 ^B^	1.12	<0.001	0.001	<0.001
Feed conversion ratio	9.57 ^A^	5.81 ^B^	7.50 ^B^	6.85 ^B^	8.73 ^A^	0.41	<0.001	<0.001	0.355
WRI (%)	9.73 ^B^	10.51 ^A^	11.89 ^A^	11.52 ^B^	6.94 ^C^	0.15	<0.001	<0.001	<0.001
ECD (%)	0.15 ^B^	0.22 ^A^	0.22 ^A^	0.20 ^B^	0.14 ^C^	0.01	<0.001	<0.001	0.001
Larval length (cm)	1.56 ^A^	1.60 ^A^	1.27 ^B^	1.48 ^B^	1.98 ^A^	0.10	0.38	<0.001	<0.001
Real FR (mg/larva/day)	101.58	101.58	57.14	114.28	133.24	1.76	0.450	0.006	0.354
SuR (%) ^(^*^)^	95.91	97.75	96.50	98.50	97.50	2.578	0.152	0.156	0.115

Abbreviations: C: Control diet, O: Omnivorous diet; RMSE: Root mean squared error; 50: feeding rate of 50 mg feed/larva/day; 100: feeding rate of 100 mg feed/larva/day; 200: feeding rate of 200 mg feed/larva/day. WRI: waste reduction index; ECD: efficiency of conversion of digested food; SuR: survival rate ^(^*^)^ see M&M. ^A–C^ Values with different superscript letters within the same line and effect are significantly different (*p* <0.01).

**Table 4:** Rearing performance and efficiency of black soldier fly larvae depending on diet (D), density (Den) and their interaction (D × Den).

	Diet (D)		Density (Den)			RMSE	*p*		
	C	O	5	10	15		D	Den	D × Den
Final larval weight (mg)	112.71 ^A^	99.69 ^B^	108.06 ^A,B^	112.63 ^A^	97.92 ^B^	0.990	0.003	0.016	0.023
Final larval biomass (g)	799.92 ^A^	717.44 ^B^	440.92 ^B^	917.13 ^A^	917.98 ^A^	4.61	<0.001	<0.001	<0.001
Final frass biomass (g)	689.88 ^A^	538.24 ^B^	404.39 ^B^	729.95 ^A^	707.85 ^A^	4.82	<0.001	<0.001	0.458
Growth rate (mg/day)	11.57 ^A^	7.82 ^B^	12.25 ^A^	9.27 ^B^	7.56 ^B^	0.128	<0.001	<0.001	0.014
Substrate reduction (%)	82.65 ^B^	86.60 ^A^	83.40 ^b^	85.01 ^a^	85.47 ^a^	0.150	<0.001	0.021	0.420
Feed Conversion Ratio	5.57 ^B^	6.42 ^A^	5.82 ^B^	5.68 ^A^	6.49 ^A^	0.054	<0.001	0.011	<0.001
WRI (%)	11.45 ^A^	9.36 ^B^	12.87 ^A^	9.14 ^B^	9.20 ^B^	0.023	<0.001	<0.001	<0.001
ECD (%)	0.23 ^A^	0.21 ^B^	0.22	0.22	0.22	0.001	<0.001	0.771	<0.001
Larval length (cm)	1.52	1.54	1.50	1.54	1.55	0.009	0.560	0.517	0.278
Real FR (mg/larva/day)	100.00	78.50	123.81	86.36	57.57	0.000	<0.001	<0.001	<0.001
SuR (%) ^(^*^)^	96.54 ^b^	98.46 ^a^	97.65	97.28	97.56	1.620	0.024	0.918	0.597

Abbreviations C: Control diet, O: Omnivorous diet; RMSE: Root mean squared error; 5: density of 5 larvae/cm^2^; 10: density of 10 larvae/cm^2^; 15: density of 15 larvae/cm^2^. WRI: waste reduction index; ECD: efficiency of conversion of digested food; FR: feeding rate; SuR: survival rate ^(^*^)^ see M&M. ^A,B^ Values with different superscript letters within the same line and effect are significant different (*p* < 0.01). ^a,b^ Values with different superscript letters within the same line and effect are significantly different (*p* < 0.05).

**Table 5:** Proximate chemical composition (% on dry matter basis unless otherwise specified) of black soldier fly larvae depending on diet (D), feeding rate (FR) and their interaction (D × FR).

	Diet (D)		Feeding Rate (FR)			RMSE	*p*		
Trial 1	C	O	50	100	200		D	FR	D × FR
Dry Matter (%)	36.71	37.39	35.85 ^B^	35.25 ^B^	40.06 ^A^	0.685	0.051	<0.001	<0.001
Crude Protein	29.97 ^B^	31.29 ^A^	32.05 ^A^	29.27 ^C^	30.56 ^B^	0.508	<0.001	<0.001	0.043
Crude Fat	20.33 ^B^	27.76 ^A^	20.37 ^C^	22.97 ^B^	28.80 ^A^	1.098	<0.001	<0.001	0.437
Ash	14.79 ^A^	6.55 ^B^	10.33 ^A^	10.37 ^A^	11.30 ^B^	0.310	<0.001	<0.001	<0.001

Abbreviations: C: Control diet, O: Omnivorous diet; RMSE: Root mean squared error; 50: feeding rate of 50 mg feed/larva/day; 100: feeding rate of 100 mg feed/larva/day; 200: feeding rate of 200 mg feed/larva/day. ^A–C^ Values with different superscript letters within the same line and effect are significantly different (*p* < 0.01).

**Table 6:** Proximate chemical composition (% on dry matter basis unless otherwise specified) of black soldier fly larvae depending on diet (D), rearing density (Den) and their interaction (D × Den).

	Diet (D)		Density (Den)			RMSE	*p*		
Trial 2	C	O	5	10	15		D	Den	D × Den
Dry Matter (%)	37.87 ^b^	39.69 ^a^	37.36 ^B^	41.02 ^A^	37.97 ^A,B^	1.761	0.045	0.006	0.354
Crude Protein	26.54 ^B^	34.67 ^A^	31.73 ^A^	30.00 ^B^	30.08 ^B^	0.477	<0.001	<0.001	0.016
Crude Fat	20.94 ^B^	27.20 ^A^	23.22 ^B^	25.57 ^A^	23.42 ^B^	0.825	<0.001	<0.001	0.140
Ash	19.11 ^A^	6.84 ^B^	12.63 ^B^	12.51 ^A^	13.78 ^A^	0.307	<0.001	<0.001	0.854

Abbreviations: C: Control diet; O: Omnivorous diet; RMSE: Root mean squared error; 5: density of 5 larvae/cm^2^; 10: density of 10 larvae/cm^2^; 15: density of 15 larvae/cm^2^. ^A,B^ Values with different superscript letters within the same line and effect are significant different (*p* < 0.01). ^a,b^ Values with different superscript letters within the same line and effect are significantly different (*p* < 0.05).

**Table 7:** Process optimization parameters for Control (C) and Omnivorous (O) diets.

	**Diet**	**FR** **(mg/Larva/Day)**	**Larval Weight (mg)**	**GR (mg/Day)**	**SR (%)**	**WRI (%)**	**ECD**	**FCR**	**Desirability**
Trial 1	C	175	128	10.4	80.8	8.41	0.12	9.59	0.542
	O	90.2	116	13.2	84.4	12.2	0.24	5.25	0.62
	**Diet**	**Den** (**Larva/cm^2^)**	**Larval Weight (mg)**	**GR (mg/Day)**	**SR (%)**	**WRI (%)**	**ECD**	**FCR**	**Desirability**
Trial 2	C	7.57	122.15	12.46	81.93	11.50	0.22	5.31	0.52
	O	5.00	108.34	11.39	86.18	12.27	0.22	5.46	0.731

Abbreviations. C: Control diet, O: Omnivorous diet; FR: feeding rate; GR: growth rate; SR: substrate reduction; WRI: waste reduction index; ECD: efficiency of conversion of digested food; FCR: feed conversion ratio; Den: density.

## Data Availability

The data presented in this study are available on request from the corresponding author.

## Supplementary Materials

The following supporting information can be downloaded at: https://www.mdpi.com/article/10.3390/insects16010005/s1. Table S1. Rearing performance of black soldier fly larvae reared applying different diet (C and O) and feeding rate (50, 100, 200) (mean values, n = 4); Table S2. Rearing performance of black soldier fly larvae reared applying different diet (C and O) and rearing densities (5, 10, 15) (mean values, n = 4).
