# Supplementary material for: Black Soldier Fly Larvae’s Optimal Feed Intake and Rearing Density: A Welfare Perspective (Part II)
Source: Insects. 2024 Dec 26;16(1):5. doi: 10.3390/insects16010005 (PMC11765738; doi:10.3390/insects16010005)
Supplement: Supplementary file 1 [file insects-16-00005-s001.zip › insects-3265805-supplementary.pdf]

Black soldier fly larvae optimal feed intake and rearing density: a welfare perspective (Part II)

Arianna Cattaneo[1], Simona Belperio[2], Luca Sardi[2], Giovanna Martelli[2], Eleonora Nannoni[2], Sihem Dabbou[1,*], Marco Meneguz[3]

[1] Center Agriculture Food Environment (C3A), University of Trento, San Michele All'Adige, 38098, TN, Italy; arianna.cattaneo@unitn.it (A.C); sihem.dabbou@unitn.it (S.D)
[2] Department of Veterinary Sciences (DIMEVET), University of Bologna, Ozzano dell'Emilia, 40064, BO, Italy; simona.belperio2@unibo.it (S.B); luca.sardi@unibo.it (L.S); giovanna.martelli@unibo.it (G.M); eleonora.nannoni2@unibo.it (E.N).
[3] BEF Biosystems s.r.l., Torino, 10156, TO, Italy; marco.meneguz@bef.bio (M.M)

Table S1. Rearing performance of black soldier fly larvae reared applying different diet (C and O) and feeding rate (50, 100, 200) (mean values, n=4).

**Diet**		**C**			**O**	
**Feeding Rate**	**50**	**100**	**200**	**50**	**100**	**200**
Final larval weight (mg)	47.53	82.33	143.49	72.12	125.17	176.64
Final larval biomass (g)	116.80	194.17	306.90	181.07	320.45	432.42
Final frass biomass (g)	173.30	358.10	546.40	94.87	260.22	524.90
Growth rate (mg/day)	3.13	8.10	9.82	6.642	14.22	12.58
Substrate reduction (%)	78.34	77.62	82.92	88.14	83.73	83.60
Feed Conversion Ratio	9.57	8.72	10.44	5.43	4.99	7.02
WRI (%)	11.19	11.09	6.91	12.59	11.96	6.97
ECD (%)	0.18	0.16	0.12	0.26	0.24	0.16
Larval length (cm)	1.20	1.42	2.04	1.35	1.54	1.91
Real FR (mg/larva/day)	57.14	114.28	133.33	57.14	114.28	133.34
SuR (%)(*)	96.00	99.00	92.75	88.14	83.73	83.59

Abbreviations C: control diet, O: Omnivorous diet; 50: feeding rate of 50 mg feed/larva/day; 100: feeding rate of 100 mg feed/larva/day; 200: feeding rate of 200 mg feed/larva/day. WRI: waste reduction index; ECD: efficiency of conversion of digested food; SuR: survival rate (*) see lines 209-210 for M&M.

Table S2. Rearing performance of black soldier fly larvae reared applying different diet (C and O) and rearing densities (5, 10, 15) (mean values, n=4).

Diet	C			O		
**Rearing density**	**5**	**10**	**15**	**5**	**10**	**15**
Final larval weight (mg)	107.48	126.17	104.49	108.64	99.09	91.35
Final larval biomass (g)	408.92	996.67	994.17	472.92	837.60	841.80
Final frass biomass (g)	465.25	819.55	784.85	343.52	640.35	630.85
Growth rate (mg/day)	13.10	12.16	9.45	11.40	6.38	5.68
Substrate reduction (%)	80.90	83.18	83.89	85.90	86.85	87.05
Feed Conversion Ratio	6.17	4.95	5.60	5.46	6.42	7.40
WRI (%)	13.48	10.40	10.48	12.27	7.89	7.91
ECD (%)	0.21	0.25	0.24	0.22	0.20	0.20
Larval length (cm)	1.50	1.57	1.50	1.51	1.52	1.59
Real FR (mg/larva/day)	133.34	100.00	66.67	114.29	72.73	48.48
SuR (%)(*)	96.41	96.05	97.16	85.90	86.86	97.97

Abbreviations C: control diet, O: Omnivorous diet; 5: density of 5 larvae/cm^2; 10: density of 10 larvae/cm^2; 15: density of 15 larvae/cm^2. WRI: waste reduction index; ECD: efficiency of conversion of digested food; SuR: survival rate (*) see lines 209-210 for M&M.
